# The Authors’ Reply

**Published:** 2013-09

**Authors:** Ali Moghtaderi, Roya Alavi-Naini

**Affiliations:** 1 Department of Neurology, Zahedan University of Medical Sciences, Zahedan, Iran;; 2 Infectious Diseases and Tropical Medicine Research Center, Zahedan University of Medical Sciences, Zahedan, Iran

**Dear Editor,**

Stroke is truly an important disease, especially because of its morbidity and mortality the world over. Wiwanitit and Wiwanitkit also confirmed this opinion and pointed out some important features of stroke in tropics in their review article published in the Journal.^1^

First of all, physicians, especially neurologists, should investigate all different etiologies of stroke in tropical regions. Indubitably for all the varieties of tropical infectious diseases, neurologists are not likely to encounter them all in the limited area where they practice. Some types of infectious diseases are, however, endemic in some tropical regions, and local physicians should have a thorough understanding of them; otherwise, the number of patients with infective etiologies of stroke will rise in those areas.^2^ Cryptogenic infections are also important causes of stroke in patients with no definite risk factors especially in young adults.^3^ Neurologists, therefore, should be well-versed in the assessment, diagnosis, and management of all these contributing factors. 

Globalization has remarkably facilitated travelling to various parts of the world within a short period of time. Unfortunately, physicians are sometimes liable to fail to inquire about their patient’s history of travelling, and patients themselves may think such details are not of any value to their physicians. However, a patient’s history of travelling to a tropical region could furnish the treating physician with a vital clue to the definitive diagnosis. Indeed, transmission of infection from an endemic to a non-endemic region is a rapidly increasing phenomenon nowadays and should be taken into consideration by all health care workers. Such awareness could safely guide general practitioners and/or attending neurologists to an early diagnosis and proper management of patients.

Finally, we fully share the opinion of the authors of the letter vis-à-vis the use of such new diagnostic modalities as novel laboratory methods and imaging procedures. Be that as it may, when I was a student of neurology, I learned that precise decision-making as regards final diagnosis requires repeated medical history taking. It goes without saying, however, that an optimal scientific and academic approach draws upon the synergy between thorough medical history taking and state-of-the-art diagnostic modalities. 
